# Characterisation of the optical response to seismic waves of submarine telecommunications cables with distributed and integrated fibre-optic sensing

**DOI:** 10.1038/s41598-024-83107-x

**Published:** 2024-12-30

**Authors:** David M. Fairweather, Max Tamussino, Ali Masoudi, Zitong Feng, Richard Barham, Neil Parkin, David Cornelius, Gilberto Brambilla, Andrew Curtis, Giuseppe Marra

**Affiliations:** 1 School of Geosciences, University of Edinburgh, Edinburgh, UK; 2 National Physical Laboratory, Teddington, UK; 3 Optoelectronics Research Centre, University of Southampton, Southampton, UK; 4 Acoustic Sensor Networks, West Sussex, UK; 5 British Telecom Applied Research, Ipswich, UK

**Keywords:** Seismology, Geophysics, Optical sensors

## Abstract

We present the first controlled-environment measurements of the optical path-length change response of telecommunication submarine cables to active seismic and acoustic waves. We perform the comparison among integrated (optical interferometry) and distributed (distributed acoustic sensing, DAS) fibre measurements and ground truth data acquired by 58 geophones, 20 three-axis seismometers and 7 microphones. The comparison between different seismic acquisition methods is an essential step towards full validation and calibration of the data acquired using novel cable-based sensing techniques. Our experimental data demonstrates broadside sensitivity of integrated optical phase measurements, in contrast to predictions from the prevailing model for this type of sensing. We also present evidence of a fast-wave arrival, which we attribute to coupled energy propagating through the metal armour of the submarine cables at a considerably faster velocity than the subsurface and acoustic waves measured during our tests. The latter process can greatly affect the detected optical signal. The experimental setup allowed us to also observe how sensing measurements on separate optical fibres within the same cable can lead to significantly different detected waveforms. Constraining the effects of the fibre architecture on recorded signals can identify factors that contribute to the non-linear response of such a sensing system.

**Supplementary Information:**

The online version contains supplementary material available at 10.1038/s41598-024-83107-x.

## Introduction

Optical fibre-based seismic monitoring is a rapidly growing discipline in Earth Science. The range of applications continues to expand and includes vertical seismic profiling^1–3^, earthquake detection^4–8^, ambient noise studies^7,9,10^, marine life tracking^11^, monitoring ocean dynamics^12^ and cryoseismology^13,14^. Most of this research has been enabled by the commercial availability of distributed acoustic sensing (DAS) fibre interrogators which can deliver metre-scale spatial resolution and a sensing range up to 150 km^15^. For optical fibre-based sensing techniques to be fully exploited in these areas of research, the transfer function between changes in the media in which the optical fibre cable is installed and changes in the properties of the light travelling in the optical fibre must be known^16^. Several conditions, such as the cable coupling to the surrounding environment, the cable geometry and armouring, as well as the fibre arrangement within the cable, can affect this transfer function. Numerous studies have sought to evaluate how each parameter contributes to the strain measured by DAS systems, particularly in response to real Earth phenomena^17–23^. Whilst the understanding of the response of terrestrial telecommunication cables and purposely designed cables for sensing applications has been improving through these studies, to the best of our knowledge, no investigation of the response of submarine cables has been performed in a controlled environment to date. A limited number of studies have been carried out to compare the cable response directly on the seafloor, but only to sparsely co-located sensors^24,25^. No previous work has directly compared the response of submarine cables to a dense, co-located ground-truth sensor array, using active seismic sources to characterise the cable response.

The understanding of the transfer function between cable perturbation and resulting changes in the optical signal can also depend on the sensing technique used. In recent years, novel techniques have shown the ability to extend the sensing range beyond that achieved by DAS, to thousands of km, at the expense of a lower spatial resolution^26,27^. These techniques have been able to detect earthquakes and ocean currents, showing the potential to extend monitoring capabilities from near-shore to the off-shore deep ocean. In these techniques, the environmentally-induced perturbations on optical parameters, such as phase or polarization, are integrated over a length of optical fibre (tens of km in the best case), in contrast with metre-scale lengths with DAS. In the case of optical interferometry^26,28,29^, the response of the cable was investigated analytically by Fichtner et al.^30^. In this model, dominant first-order effects are generated from inline deformation of the fibre whereas perpendicular deformation generates only second order effects whose amplitude is several orders of magnitude smaller. The model predicts that, for sources perpendicular to the cable, environmentally-induced changes in the measured optical phase arise exclusively from fibre bends, if these are present, and from the optical path discontinuity at the ends of the cable. If this prediction holds, and there is no first-order broadside sensitivity for integrated measurements, then this could be a considerable limitation for optical interferometry applied to the existing submarine telecommunications network. One significant consequence of this is that localisation of seismic wave arrivals, as illustrated in^28^, would not be possible.

Submarine cables are significantly different from telecommunication cables installed on land or used in dedicated DAS installations. They are typically heavily armoured near the coast where the risk of damage, usually from ship anchors and fish trawlers, is greater. At water depths greater than 1500 m, where the risk is lower, the armouring is typically reduced. However, even in the case of lightly armoured submarine cables (commonly referred to as Light Weight cables, LW) optical fibres are installed within rigid copper tubing and surrounded by layers of steel wires, thus significantly more heavily armoured than telecommunication cables used on land. This could lead to differences in how external mechanical changes propagate to the optical fibre inside the cable.

Here, we perform the first cable response measurements of submarine cables in a controlled environment. We simultaneously measure the changes in the optical fibre with both integrated (optical interferometry) and distributed (DAS) techniques on optical fibres within the same cable and compare them with ground truth data acquired with 1- and 3-component seismometers, as well as acoustic data from microphones. Additionally, both DAS and interferometric measurements were performed on two adjacent fibres within each cable. These tests led to three main conclusions: (1) Our experimental evidence indicates first-order sensitivity of interferometric measurements on fibre laid straight to perpendicular sources. Hence, we do not find experimental confirmation of the model proposed by Fichtner et al.^30^; (2) We show that the cable armour can play a considerable role in the measured optical signals, by channelling and propagating energy within the cable at higher speeds than through the ground—a phenomenon we call the ‘fast wave’; (3) We observe considerable differences in the waveforms detected by adjacent fibres within the same cable, which also appear to be dependent on the interrogation technique used.

## Experiment

### The test bed

**Fig. 1:**
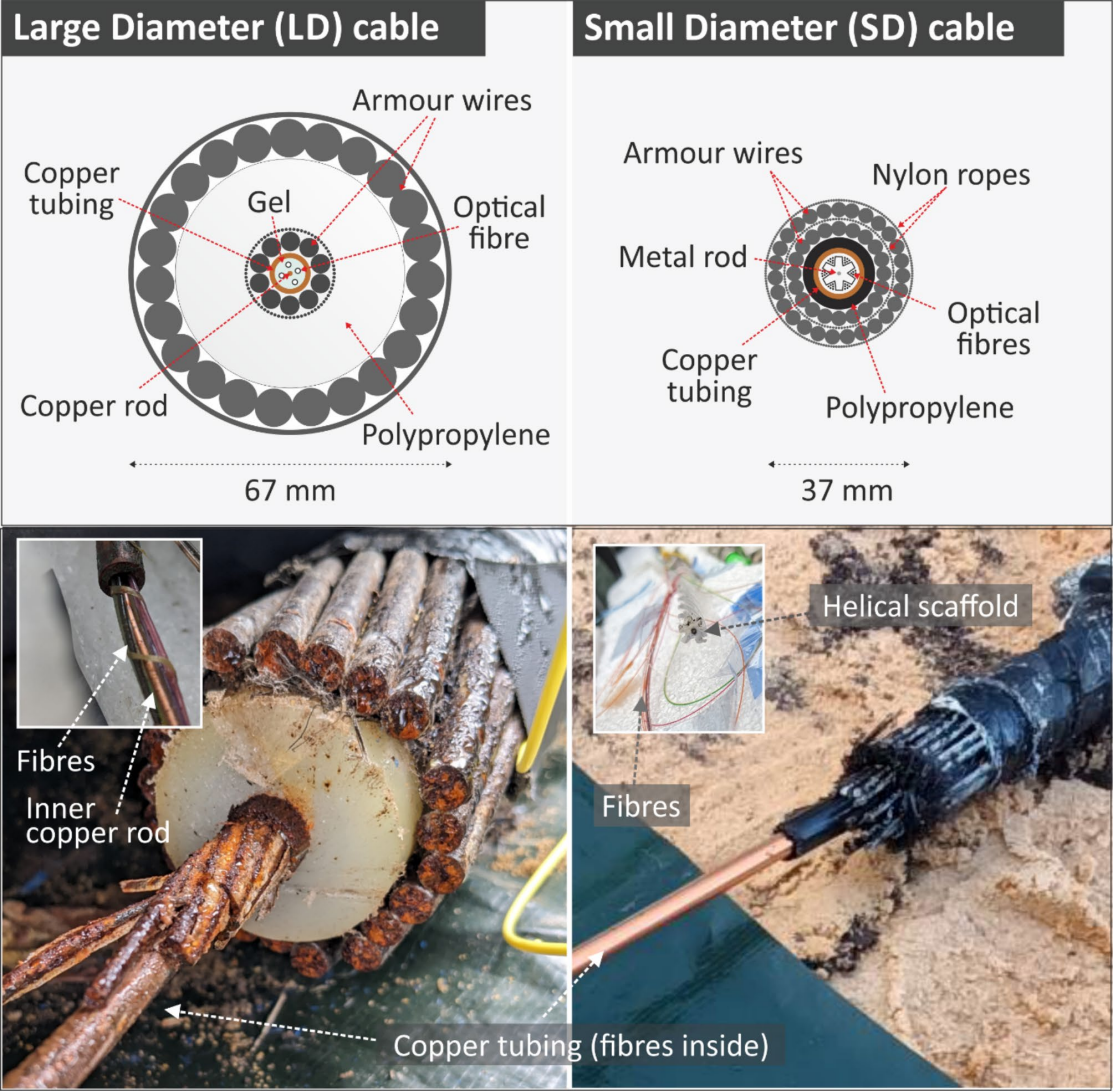
Cross section and photographs of the LD and SD submarine cables under test. Details of the fibres emerging from the protecting copper tubing is shown in the inset, including the helical plastic scaffolding supporting the fibres in the SD cable.

The experimental test bed consisted of two telecommunication submarine cable samples with different armour structures and an un-armoured optical fibre, protected by a 2 mm-diameter plastic jacket. The cross section of the two submarine cables is shown in Fig. 1. In the text that follows, we will refer to the Small Diameter and Large Diameter cables as SD and LD respectively, and the jacketed fibre as JF. The length and outer diameter of the SD and LD cables are 26 m and 37 mm, and 24 m and 67 mm respectively. The length of the JF is 26 m. The two submarine cables present differences in the number of armouring layers of steel wire and overall structure, as shown in Fig. 1. Both cables have two layers of steel wire armour. However, the two layers are separated by a thick layer of polypropylene in the LD cable whilst in the SD cable only by a thin layer constituting nylon ropes. The SD cable has 24 optical fibres helically wound around a central plastic scaffold, organised in groups of 6. The winding direction is periodically inverted along the cable. The LD cable has 4 fibres loosely installed within the copper tubing, without a central scaffold. In both cables the space between the fibres and the copper tubing inner surface is filled with a viscous gel and a central metal rod runs in the middle of the copper tubing. We believe the fibres in the LD cable also follow a helical stranded arrangement, but it was not possible to directly verify this during our tests.

**Fig. 2:**
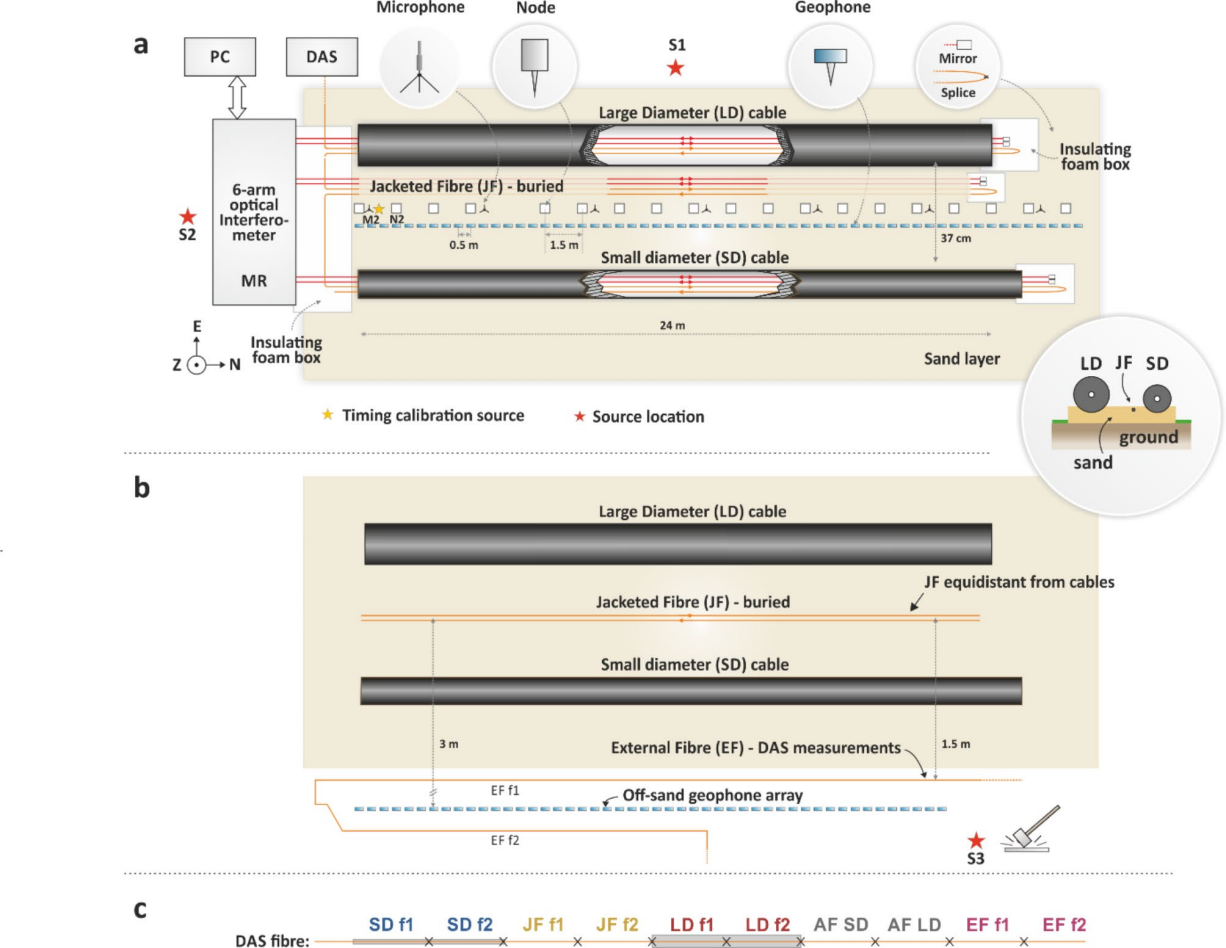
(**a**) Schematic of the measurement setup (not to scale). Optical fibres interrogated by the 6-arm interferometer are coloured red; Fibres used for DAS measurements are coloured orange and are spliced together forming a single optical path. MR: measurement rack; PC: computer used for the acquisition of interferometer and microphone data; S1, S2 and S3: source locations; (**b**) Illustration of the setup used for investigating the fast wave phenomenon. JF was rearranged equidistantly from the LD and SD cables, and an additional DAS fibre section (EF) and a geophone array were installed off-sand. (**c**) Illustration of the different sections of the fibre interrogated by the DAS system. Crosses indicate the splice points. AF refers to fibre attached directly to the armour, which is discussed later.

The full test bed used for the cable response measurements is shown in Fig. 2. The armoured cables were both placed upon an approximately 10 cm-thick layer of sand and at an average spacing between them of 37 cm. The sand layer was intended to be an approximate analogue for unconsolidated sediment on the seafloor and, due to precipitation in advance and during the tests, its moisture content was high. The JF was placed alongside the SD cable at less than 5 cm and buried a few centimetres in the sand. During the installation of the submarine cables on the sand every possible effort was made to lay them in a straight line.

### Optical interferometry and DAS measurements setup

A total of four optical fibres were used in each cable for the measurements: two for optical interferometry and two for DAS. This arrangement was chosen to enable a first evaluation of the level of agreement between detected signals within the same cable. Additionally, it also allowed us to easily identify the beginning and end of each fibre under test in DAS measurements, as a specular pattern of the environmental perturbations is generated. A single interrogator was used for DAS measurements and the fibre pairs in the SD, LD cables and the JF were spliced together to form a single optical path for a total length of 290 m. The interferometric measurements were performed using a 6-arm optical interferometer (Fig. SM1) and connected to each fibre pair of the SD and LD cables and the JF. At the end of each fibre used for the interferometric measurements a fibre-pigtailed Faraday rotator mirror (FRM) was installed to retroreflect the optical signal. The interferometer was driven by a commercial narrow-linewidth laser source (RIO Orion). The accumulated optical phase changes on each fibre length were measured by a phase meter at a sampling rate of 1000 samples per second (Sample/s). The interferometer and other devices required for the tests were installed in a Measurement Rack (MR) within an anti-shock flight case to minimise vibration sensitivity to the environment. A polyester canopy covering the flight case, the submarine cable ends and the interconnecting fibres were installed to reduce unwanted noise arising from wind-induced movements of the interconnecting fibres between the flight case and the cables. Whilst the high spatial resolution of DAS measurements allows for sections of the optical fibres outside the test area to be ignored in post-processing, this was not possible for measurements with the optical interferometer and perturbations between the equipment rack and the test bed needed to be minimised. At the opposite end of the cables with respect to the measurement rack, the exposed optical fibres and optical components (FRMs) were installed in foam boxes to reduce sensitivity to acoustic noise and vibrations. The overall test bed was sheltered from lighter winds by trees on three sides of the test site, with only the west side in Fig. 2 being exposed. A DAS interrogator unit, developed at the University of Southampton^31^, was placed inside a van parked 8 m away from the test bed. Tests showed negligible direct pick up of the acoustic and vibrational energy of the mallet and plate source shots by the DAS unit. The gauge length of DAS measurements was set to 1 m with a spatial sampling of 33 cm and a sampling frequency of 50 kSample/s.

### The seismic and acoustic instrumentation

**Fig. 3:**
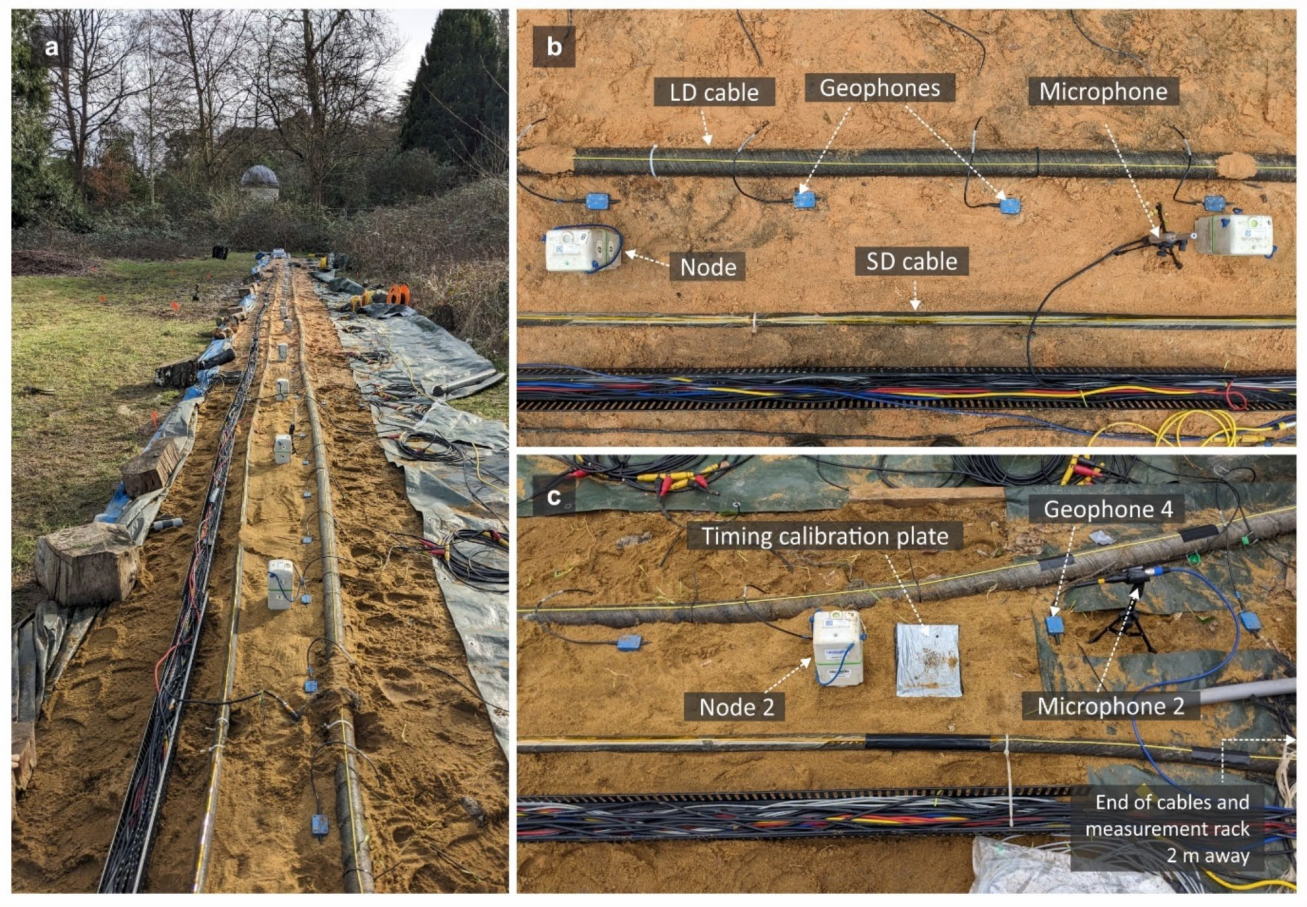
(**a**) View of the test bed from the MR end. For many of the testing sessions, the ground was saturated due to considerable precipitation in preceding days. (**b**) Close-up photo of cables and seismic and acoustic sensors. (**c**) Location of the timing calibration plate near node 2, close to the MR end of the cables. Here the two submarine cables diverge slightly. Their ends are just off the picture, extending approximately a further metre in length.

Several seismic and acoustic sensors were installed in close proximity to the cables and JF under test. Twenty seismic nodes (SmartSolo IGU-16HR 3C) were spaced 1.5 m apart on the sand between the cables and recorded three component velocities – vertical (Z) and horizontal (N, E). In these tests we aligned the positive N component along the direction of the cable and away from the MR, as shown in Fig. 2. The positive E direction therefore pointed to the right of the cables looking away from the MR. Up to 58 vertical component geophones (Geospace GS20DX) were used to sample the array at a higher spatial resolution with an interval of 0.5 m. Seven omnidirectional microphones mounted on small tripods were also installed at 4.5 m spacing along the cable. Figure 3a shows a photo of the testbed, and Fig. 3b shows details of the seismic and acoustic measurement equipment along the cables.

### Seismic source and measurement procedure

The active sources consisted of sledgehammer blows (aka. shots) on a steel plate at selected locations around the testbed, three of which are represented by red stars in Fig. 2. These included inline shots from which the recorded wavefield was assumed to have travelled dominantly in the direction of the cables, and perpendicular shots from which the wavefield was incident on one side of the cables. In Fig. 2 only a subset of the tests is shown; more tests are shown in the Supplementary Materials. For each test, one timing calibration shot was struck on a smaller steel plate located equidistantly from each submarine cable (Fig. 3c), the second node N2, geophone 4, and approximately 35 cm from the second microphone M2 (numbering starts from the MR end). This provided a common source to calibrate timestamps across geophones, nodes, DAS, interferometer and microphones. This was required because while nodes were GPS-timed, the geophones, DAS data, and microphones/interferometer data were recorded using separate devices. The first arrivals from each instrument were aligned to the first arrival waveform on the closest node, and data were synchronised to an uncertainty of 1 ms. The seismic instrumental responses were removed from the measurements and units of the data were converted to displacement. The interferometric measurements are expressed as phase data and the DAS data as strain.

## Results

### Broadside sensitivity of integrated measurements

**Fig. 4:**
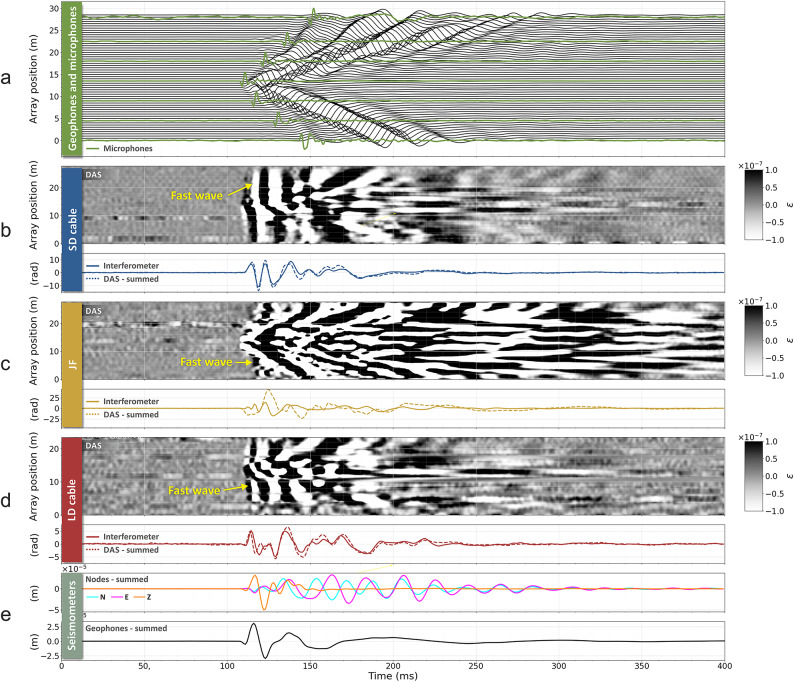
Data comparisons for the perpendicular source location S1. All data are band-passed from 10–300 Hz with a sixth-order Butterworth filter. DAS channels are represented by the greyscale plots of strain *ε* for the SD cable, JF and LD cable. The timeseries below each colourmap show the respective summed DAS channels and interferometric measurements for the same segment; summed seismic receivers are shown in the bottom two traces.

The results of a test in which the seismic source (S1) was located 1 m away from the centre of the testbed and perpendicularly to the cables are shown in Fig. 4. Panel (a) shows the waveforms recorded by the geophones alongside those from microphones (green traces). Panels (b), (c) and (d) show the DAS and interferometer signals for the SD and LD cables and the JF. Each of these panels shows a plot of DAS data and the total optical phase change, measured by summing all DAS channels, in comparison to the phase changes measured by the optical interferometer. Panel (e) shows the waveforms resulting from summing the signals from the geophones and from the nodes. Good agreement is found between the summed DAS and the interferometer waveforms for the LD and SD cables.

We find that the agreement is considerably worse for the JF. This is expected as JF consisted of two 30 m-long duplex patch cords buried together in the sand. Whilst every effort was made to ensure the two patch cords run together, this arrangement is likely to lead to a substantially lower degree of agreement of the detected signals compared to adjacent fibres within the same cable, which is more prone to differences in local coupling to the ground. Another contribution arises from differing paths of the DAS and interferometer duplex fibres at the ends of the cable. In particular, the interferometer JF rises from the sand into the foam box (containing also the SD cable Faraday mirrors), making it more susceptible to external perturbations. The impact of this is particularly visible for sources located at the ends of the cable, as shown in Fig. SM5, where the DAS and interferometer traces differ considerably for the JF.

**Fig. 5:**
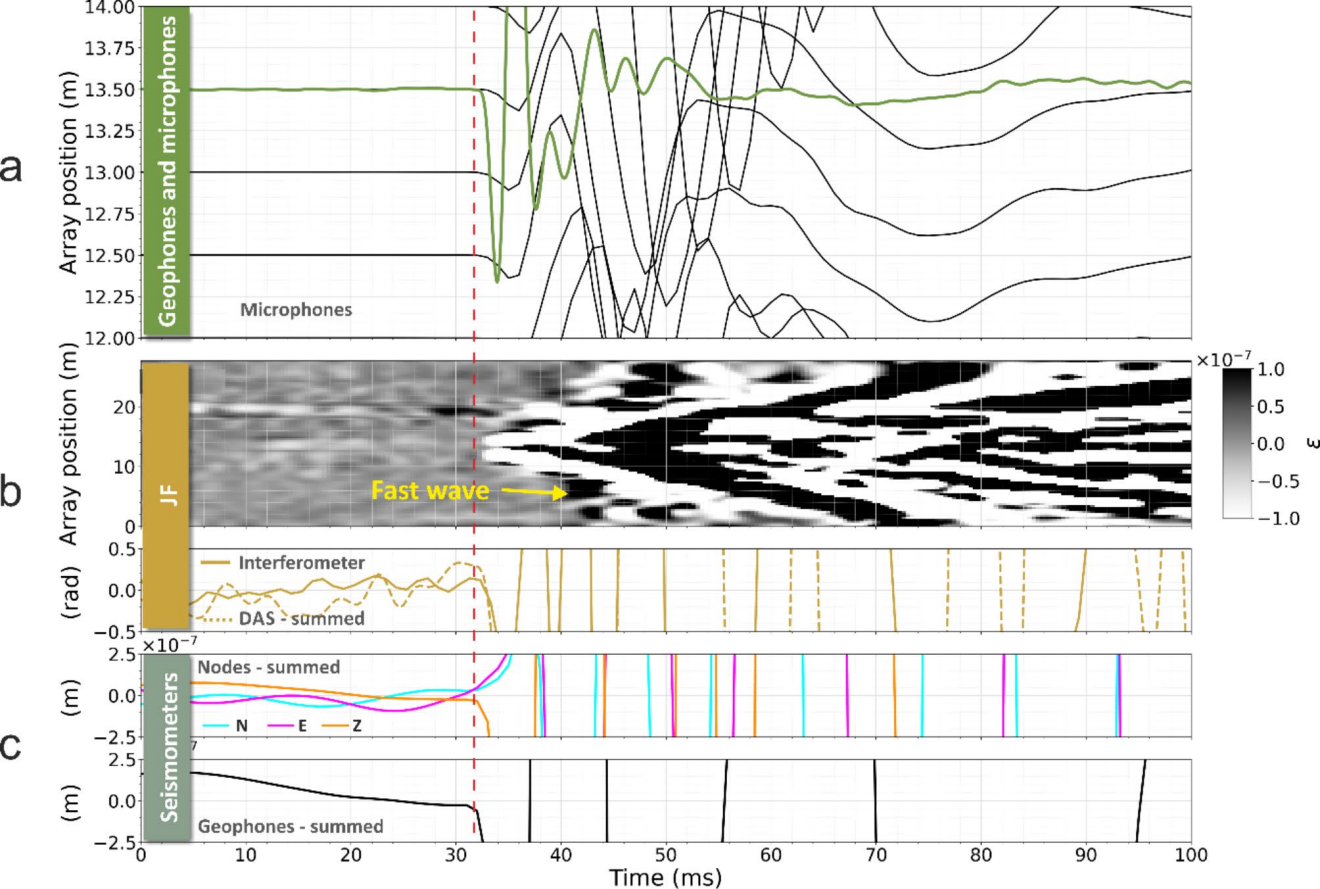
Magnified view of the JF data from Fig. 4, to more easily observe the sudden optical phase change detected by the interferometric measurement corresponding to the seismic wave arrival on the nodes and geophones. All data have been band-passed from 10 - 300 Hz with a sixth-order Butterworth filter. The optical phase recorded by the interferometer shows changes concurrently with signals from seismometers, as highlighted by the dashed red line. The geophone traces in the top panel have been locally normalised.

An important outcome of this perpendicular-source test is that a change in the measured optical phase is detected in the interferometric measurements concurrently to changes recorded by the nodes/geophones and DAS. This appears to contrast with the model proposed by Fichtner et al.^29^ which concludes that in interferometric measurements a signal should only be observed when the seismic wave reaches the ends of the cable or at bend locations (if present). In our tests, the first change in the optical phase detected by the interferometer is observed approximately 10 ms earlier than the earliest arrival at the cable ends. The very mild bends due to the ground conformation and the cable stiffness in our test bed (Fig. 3a) do not constitute a concern for the validity of these tests. Optical fibre bends due to the helical winding within the submarine cables also cannot be responsible for their broadside sensitivity, since this result is also observed in the JF, which was straight. In Fig. 5 we show a magnified view of the arrivals for the JF, to better show the onset of an optical phase change well in advance of the seismic wave reaching the ends of the fibre. In Fichtner’s model, for a straight fibre and perpendicular source, a signal might be observed due only to second-order effects. For a harmonic deformation of the cable, frequency doubling of the detected interferometric signal is expected^30^. However, we observe no frequency doubling in our tests. As shown in Fig. 4 and SM4, we observe components of same fundamental frequency as detected by nodes and geophones. We also observe no significant change in the detected signals arising from the discontinuity at the cable ends.

### Fast wave mechanisms

**Fig. 6:**
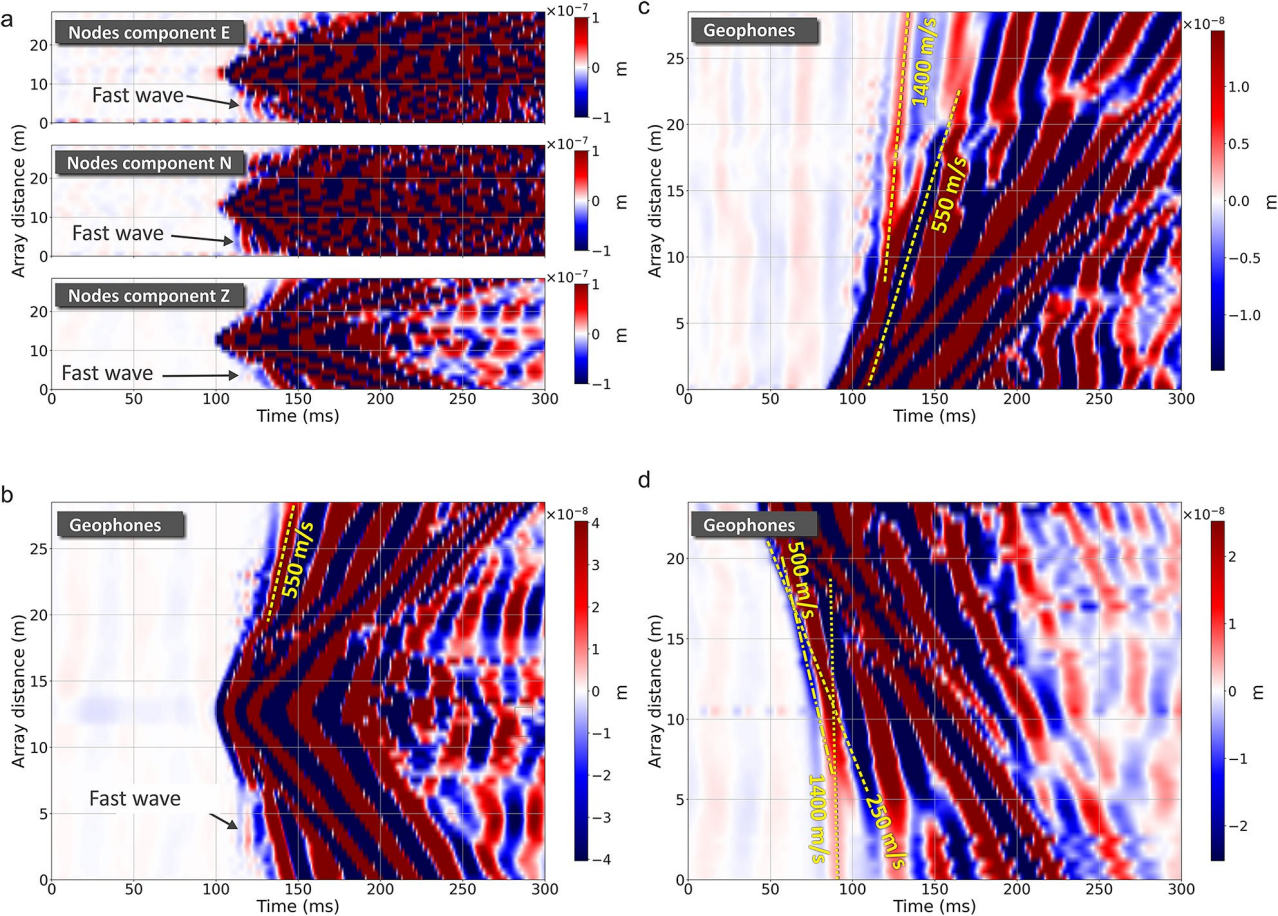
(**a**,** b**) Colourmap plot of the node and geophone array data with amplitudes clipped at a level 200 times smaller than the largest amplitude, to bring out the fast wave signal. (**c**,** d**) Colourmap plot obtained from the off-sand geophone array for in-line tests S2 and S3 respectively. The moveout lines indicate velocities considerably lower than that of the fast wave observed in DAS data for SD and LD cables.

From the DAS recording on the SD and LD in Fig. 4 we observe an almost-simultaneous arrival across all channels. The analysis of the gradient of this wave across different tests leads to a seismic velocity in excess of 3500 ms^− 1^ (the *fast wave*). This is not readily observed in the individual geophone traces on the same plots but can be seen in all components of the seismic instrument data when plotted as a similar colourmap with amplitudes clipped by two orders of magnitude (Fig. 6a,b).

We investigated two possible propagation mechanisms that could be responsible for the observed fast wave. Firstly, we hypothesized the existence of an extremely fast subsurface layer. The test site was previously used for construction materials storage and handling and several pieces of concrete protruding from the ground surface could be found in various locations. We speculated whether the possible composition of hard materials in the subsurface could provide a coherent wave energy conduit at the appropriately high velocities. However, data from the geophone array obtained from the in-line shot S2 showed that the fastest ground velocity recorded is 1400 ms^− 1^ (Fig. 6c), compatible with expectations for ground that is heavily saturated from precipitation, but still considerably slower than the observed fast wave. The velocities calculated from the 48-geophone line with 0.5 m spacing off the sand (Fig. 2b) agreed (Fig. 6d). For the perpendicular source apparent velocities agree with the slower phase of 550 ms^− 1^, and even when correcting for the offset geometry of the source we observe no velocities sufficiently high to explain the fast wave observations.

**Fig. 7:**
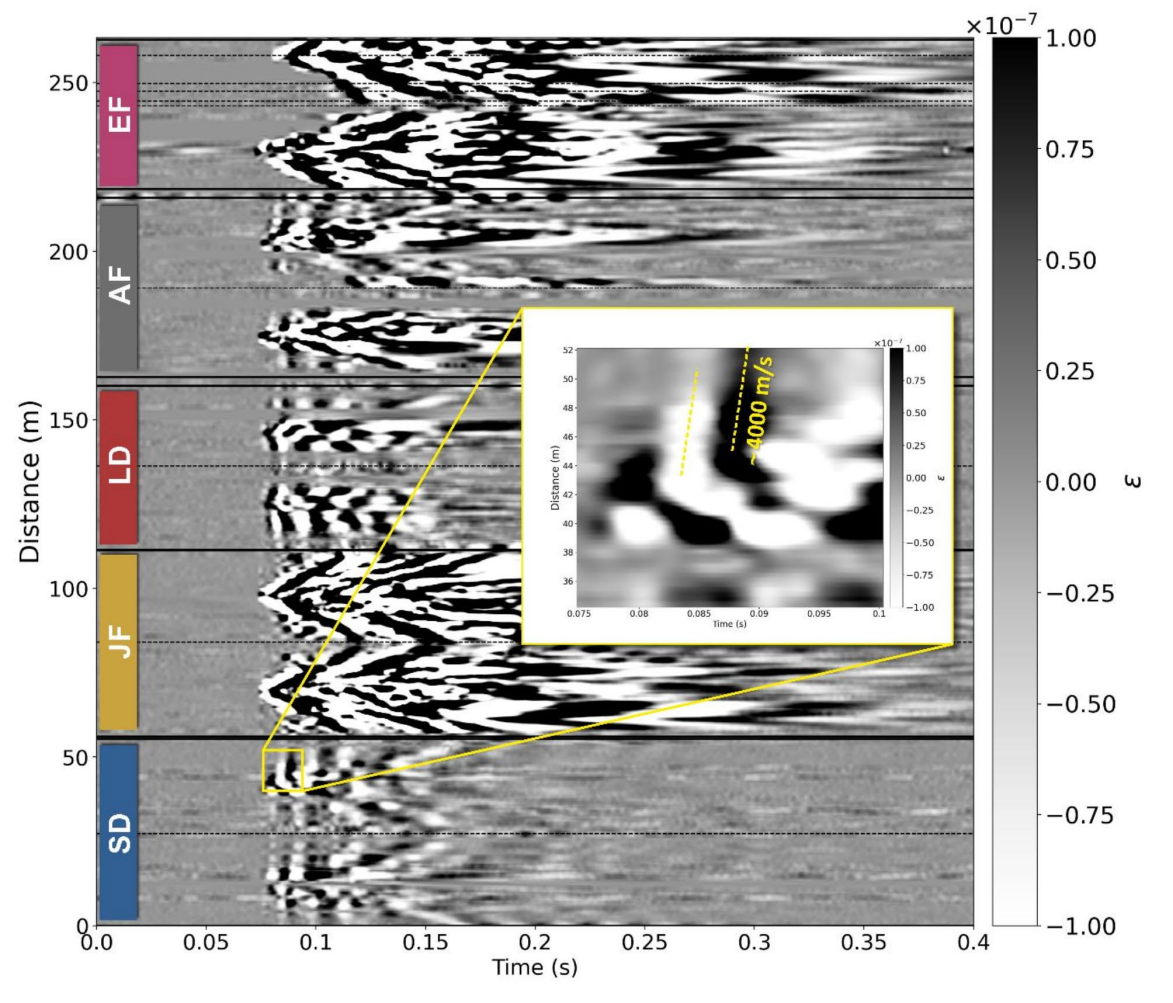
Waterfall plot of DAS strike 1 data at source location S1. Annotated fibre sections are delineated by solid lines and dashed vertical lines indicate the turning points between out and in sections, except for the extra fibre (EF) section that has additional turning points as shown in Fig. 2b. Note that the EF section does not contain these fast arrivals. The magnified inset shows the fast wave as seen on the small cable (SD) and the dashed line corresponds to a velocity of > 3000 ms^-1^. AF: additional fibre attached to the outside of the SD and LD cables.

Hence, we investigated a second hypothesis of faster propagation of seismic energy along the submarine cable through its metal armour^32^. In Fig. 4 we note that the fast wave is also observed on the JF, which would initially rule out the hypothesis that the fast wave is due to the armour. However, the JF is buried just a few centimetres away from the SD cable and so energy travelling within the cable armour could potentially be emitted and couple to the JF through the sand. To verify this possible mechanism, we extended the DAS sensing line by installing an additional fibre section (which we will call External Fibre, EF) off the sand and proximal to the off-sand geophone line, as shown in Fig. 2b. This fibre was lightly covered with sand to improved coupling to the grass surface. Furthermore, the seismic equipment was removed from the centre of the array and the JF was replaced equidistantly between the armoured cables, thus increasing the distance from the SD cable, to test whether the fast wave was still observed. Figure 7 shows all DAS fibre segments for one strike of a test performed from the source location S1. Whilst the fast wave is apparent on the SD and LD cables, it cannot be observed on the EF placed off the sand. We note that the fast wave is still visible on the JF, although with lower intensity, supporting the hypothesis that the fast wave observation is due to coupling to the SD through the sand.

Since the fast wave is observed across the armoured cable DAS data and in the JF, we conclude that its most likely source is seismic energy coupling to the cable and travelling through the metal armour. As shown in these tests, this mechanism can greatly change the resulting waveforms of the detected signal. We note that in the DAS data for the SD and LD cables in Fig. 4, some moveout consistent with that observed on JF can be identified. However, the fast wave appears to have higher intensity. We also note that the measured speed of the fast wave is lower than the typical propagation speed expected in steel (5 to 6 km/s). Several factors can influence this measured value. For example, the helical arrangement of the steel wires of the cable armour leads to a lower apparent speed with respect to a straight-line arrangement. Also, the propagation speed can be influenced by the specific modes of propagation through the heterogeneous material composition and complex structure of the cable. Indeed, we note that our measured value of agrees with previously reported propagation speeds in cable armours in^32^. The outcome of these tests suggests that the possible influence of the armour on the cable response to environmental perturbations can be considerable and should be considered and further investigated in future seafloor cable-based sensing tests. This might be especially true in near-coastal tests, where heavily armoured cables are used. We note that no lightweight cable was available for these tests. However, whilst their armouring would be much lighter than in the near-shore cables tested here, their optical fibres are still enclosed in a copper tube surrounded by steel wires, so effects similar to those shown in this work might still take place. Further research is needed to establish whether the phenomenon plays a role also at source frequencies higher and lower than investigated here.

### Waveform agreement between adjacent fibres

**Fig. 8:**
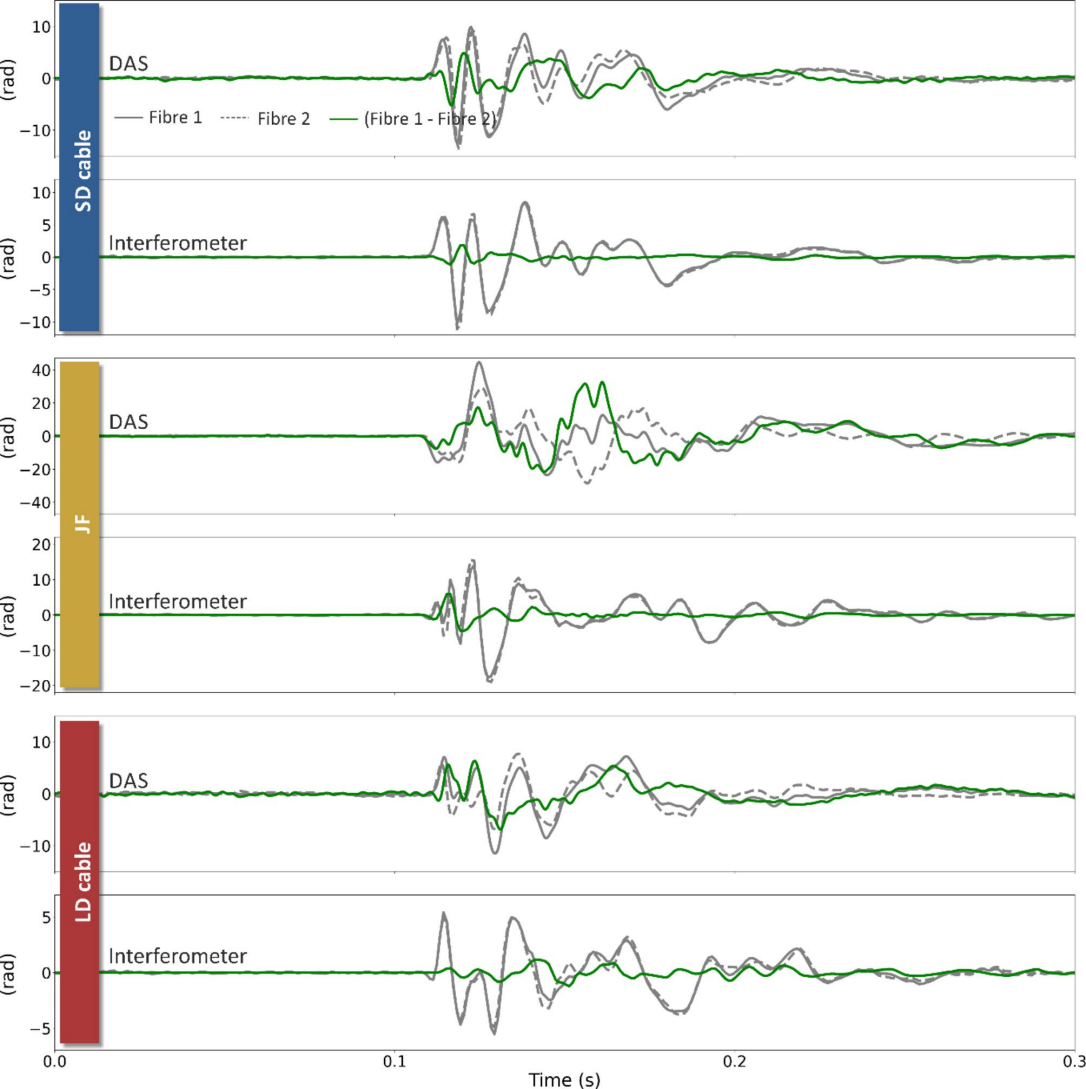
Comparison traces for each fibre segment for the summed DAS channels and the interferometer measurements. The grey solid trace is fibre 1 (f1) and the grey dashed trace is fibre 2 (f2); the green trace shows the difference between the two. The source is the same as that shown in Fig. 4. The data have been band-passed from 10–300 Hz with a sixth-order Butterworth filter.

By using two optical fibres for each type of measurement (DAS and interferometry) and each cable (SD, LD and JF), we were able to investigate the degree of agreement between waveforms from co-located fibres. Each panel of Fig. 8 shows the waveforms detected on each fibre pair, as well as the difference between them. We show this for each cable for measurements from both the DAS and interferometer. We observe substantial differences between the waveforms detected by each fibre of each fibre pair. We note that DAS measurements consistently appear to provide higher discrepancies. We explored whether the observed discrepancy could originate from signal cycle-skipping during acquisition. Indeed, should the optical path change be larger than π between DAS samples, the additional signal cycles would not be tracked, leading to waveform distortion. However, the higher level of discrepancy of DAS measurements with respect to interferometric measurements is observed also at lower intensity shots (SM5), thus not supporting this hypothesis. Another source of the discrepancy could be attributed to the non-linear contribution to the total phase change measured by a single-frequency DAS system^33^. This error arises from inherent inhomogeneities in the sensing fibre and can range from - π to π. The green traces on Fig. 8 show amplitudes comparable to this for the SD and LD cables. This can be reduced by implementing a multi-frequency DAS acquisition (see Fig. 3a from^33^) and this is a potential avenue for future tests required to better characterise sources of discrepancy with DAS measurements. However, whilst the interferometric measurements show a better degree of similarity, differences are still not negligible between fibres within the same cable. These measurements show that local conditions can play a considerable role in the detected optical path length changes. Even in the case of the LD and SD cable, where optical fibres share the same confined space, discrepancies up to 40% of the highest detected amplitude are observed. A better degree of similarity should be expected for lower frequencies than those tested here, and much higher for very slow processes such as temperature changes.

## Conclusions

The tests performed in this work are the first characterization of the optical path-length response of submarine cables to external mechanical perturbations performed in a controlled environment. The optical changes were measured simultaneously with two different sensing techniques: optical interferometry and DAS. By comparing the optical signals with seismic and acoustic data, and correlating first arrivals across instruments, we were able to demonstrate broadside sensitivity of interferometric measurements, in contrast with results expected from the prevailing model for this type of measurement. We conclude that integrated measurements can detect seismic wave arrivals from perpendicular sources. This should also enable localisation of arrivals along the cable by interrogating it from both ends, as illustrated in^27^. Our results also showed a fast-wave process taking place in measurements on submarine cables, which we attribute to coupled energy travelling through the metal armour. This appears to make a substantial contribution to the detected waveforms. Lastly, we showed that considerable differences in the detected waveforms can be observed even in the case of two optical fibres installed within the same cable. The observed difference appears to be larger for DAS than interferometric measurements and more research is needed to investigate this. These first controlled-environment measurements constitute a necessary step for a better understanding of how the optical fibres within submarine cables respond to environmental changes and towards achieving a calibrated transfer function. This is crucial in order to be able to fully exploit the diverse possibilities offered by fibre sensing technologies for Earth science research when applied to subsea cable infrastructure.

## Electronic Supplementary Material

Below is the link to the electronic supplementary material.

Supplementary Material 1

## Data Availability

All data are available from the corresponding authors upon request.
