# Supplementary material for: Characterisation of the optical response to seismic waves of submarine telecommunications cables with distributed and integrated fibre-optic sensing
Source: Sci Rep. 2024 Dec 30;14:31843. doi: 10.1038/s41598-024-83107-x (PMC11685897; doi:10.1038/s41598-024-83107-x)
Supplement: Supplementary file 1 — Supplementary Material 1 [file 41598_2024_83107_MOESM1_ESM.docx]

**Supplementary Material**

**Characterisation of the optical response to seismic waves of submarine telecommunications cables with distributed and integrated fibre-optic sensing**

David M. Fairweather^1,2^*, Max Tamussino^2^, Ali Masoudi^3^, Zitong Feng^2^, Richard Barham^4^, Neil Parkin^5^, David Cornelius^5^, Gilberto Brambilla^3^, Andrew Curtis^1^ and Giuseppe Marra^2^*

^1^ School of Geosciences, University of Edinburgh, Edinburgh, UK

^2^ National Physical Laboratory, Teddington, UK

^3^ Optoelectronics Research Centre, University of Southampton, Southampton, UK

^4^ Acoustic Sensor Networks, West Sussex, UK

^5^ British Telecom Applied Research, Ipswich, UK

* Corresponding authors. Email: david.fairweather@ed.ac.uk, giuseppe.marra@npl.co.uk

**1. 6-arm Interferometer**

The figure below shows the 6-arm interferometer used for measuring the environmentally-induced phase changes in 2 fibres per type of cable (LD, SD and JF). The light from a narrow-linewidth laser (RIO Orion) is split into two optical arms. One of the two arms is split again 6 ways and each output is sent to each fibre under test. At the end of each fibre under test, a Faraday Mirror (FM) retroreflects the light back into the same fibre and the returned light is extracted with a power splitter. The second arm is frequency shifted using an Acousto Optic Modulator (AOM) and then split 6 ways. The AOM is driven with 40 MHz from a signal generator. Each frequency shifted output is combined with the returned light from each FM onto a Photo Detector (PD), generating a beat at the AOM frequency. The phase changes on each beat frequency are then measured with a multi-channel synchronous phase meter and logged with a PC. The phase meter and the signal generator share a common reference.

**
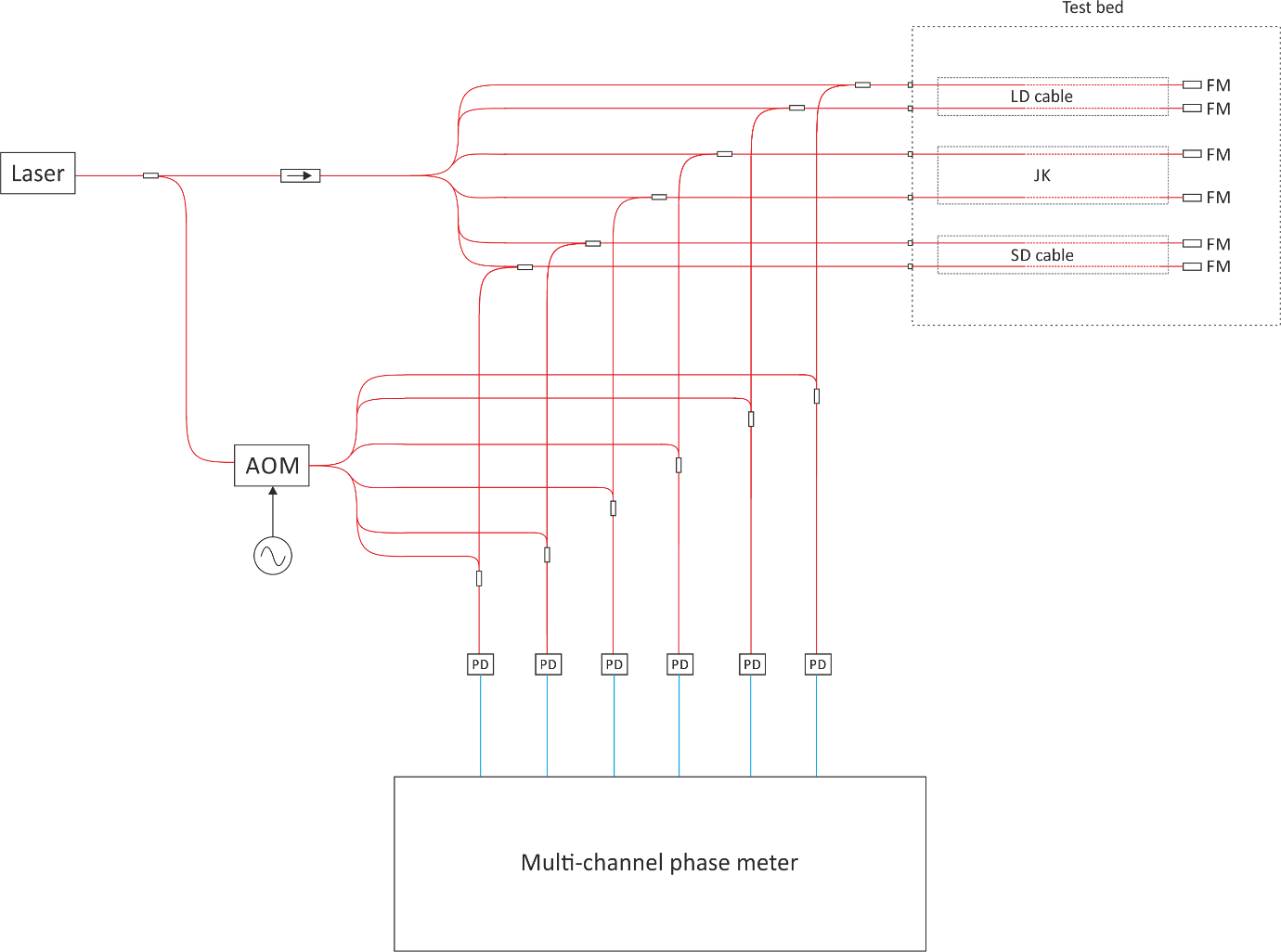
**

**Figure SM1**. The 6-arm interferometer.

**2. Convention for source location naming**
In these supplementary materials we present additional tests from a variety of source locations and we will adopt a convention for these as NX_YL, where NX denotes the nearest node number to the source and Y is the distance in metres away from the centre of the testbed. L refers to the left side of the testbed when looking down the array from MR, Fo refers to forward and Re refers to reverse. Node 1 is the closest to MR. As simpler naming choice was chosen for the main text as only 3 tests were shown. With reference to fig. X in the main text, we will have the following equivalence:

| Figure X name | Supplementary materials convention |
| --- | --- |
| S1 | N9N10_1L |
| S2 | N1_3Fo |
| S3 | N17_4.5L |
| S4 | N14_1L |

**3. Timing calibration shot**


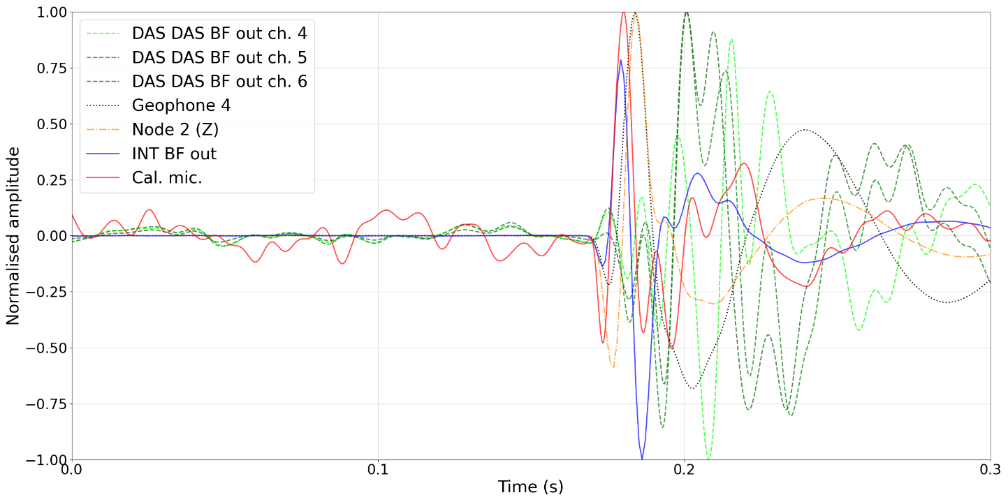


**Figure SM2**. Calibration shot for the test in Fig. 3 - the calibration shot location is at 1.5m down the array, on a small steel plate on the sand between the SC and BC equidistant from node 2, geophone 4, the calibration microphone and the BF. The first arriving energy above the background noise is taken to be the time of arrival from this shot. The 3 DAS channels cover the 1.5 m array position of the calibration shot and the amplitudes have been normalised for the purposes of this alignment.

**4. Cable response tests - additional source locations**

**N20_1Re**


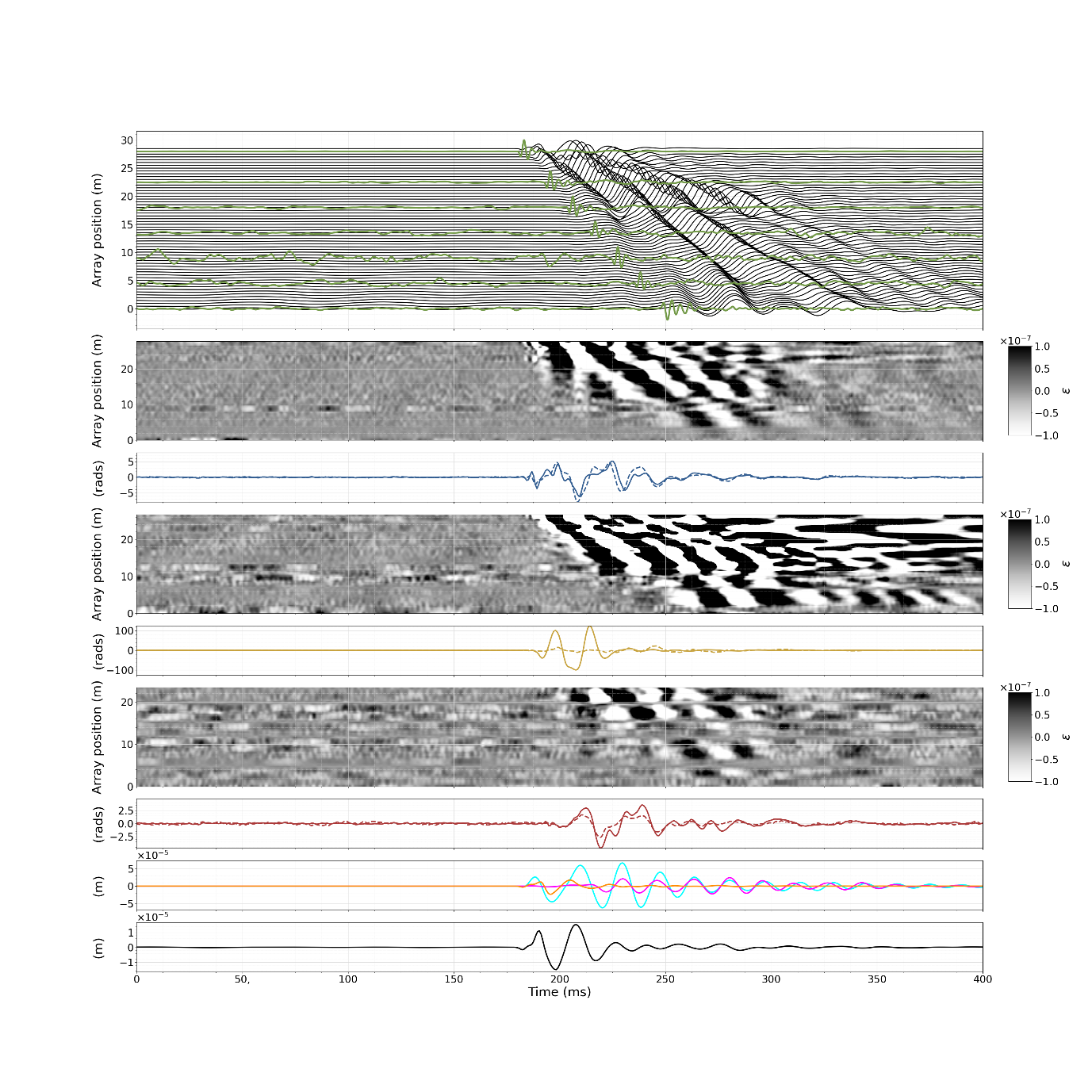


**Figure SM3**. Data comparisons for the reverse inline source location N20_1Re. All data are bandpass filtered from 10-300 Hz. DAS channels are represented by the greyscale plots for the SD cable, JF and LD cable. The timeseries below each colourmap show the respective summed DAS channels and interferometric measurements for the same segment; summed seismic receivers are shown at in the bottom two traces.

**N14_1L**
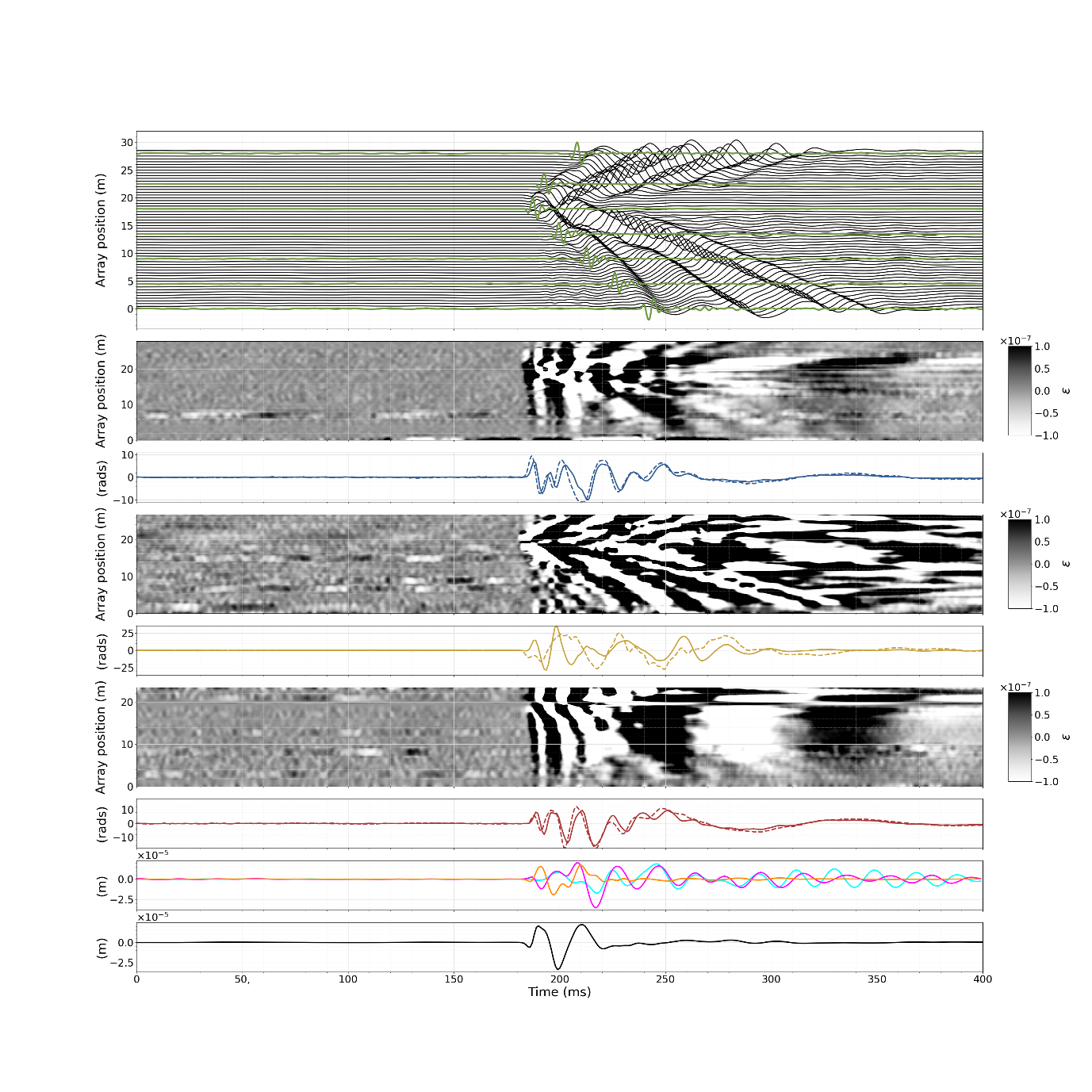


**Figure SM4.** Data comparisons for the offset perpendicular source location N14_1L. All data are bandpass filtered from 10-300 Hz. DAS channels are represented by the greyscale plots for the SD cable, JF and LD cable. The timeseries below each colourmap show the respective summed DAS channels and interferometric measurements for the same segment; summed seismic receivers are shown at in the bottom two traces.

**5. Difference between adjacent fibres with decreasing strike intensities**

Strike 1


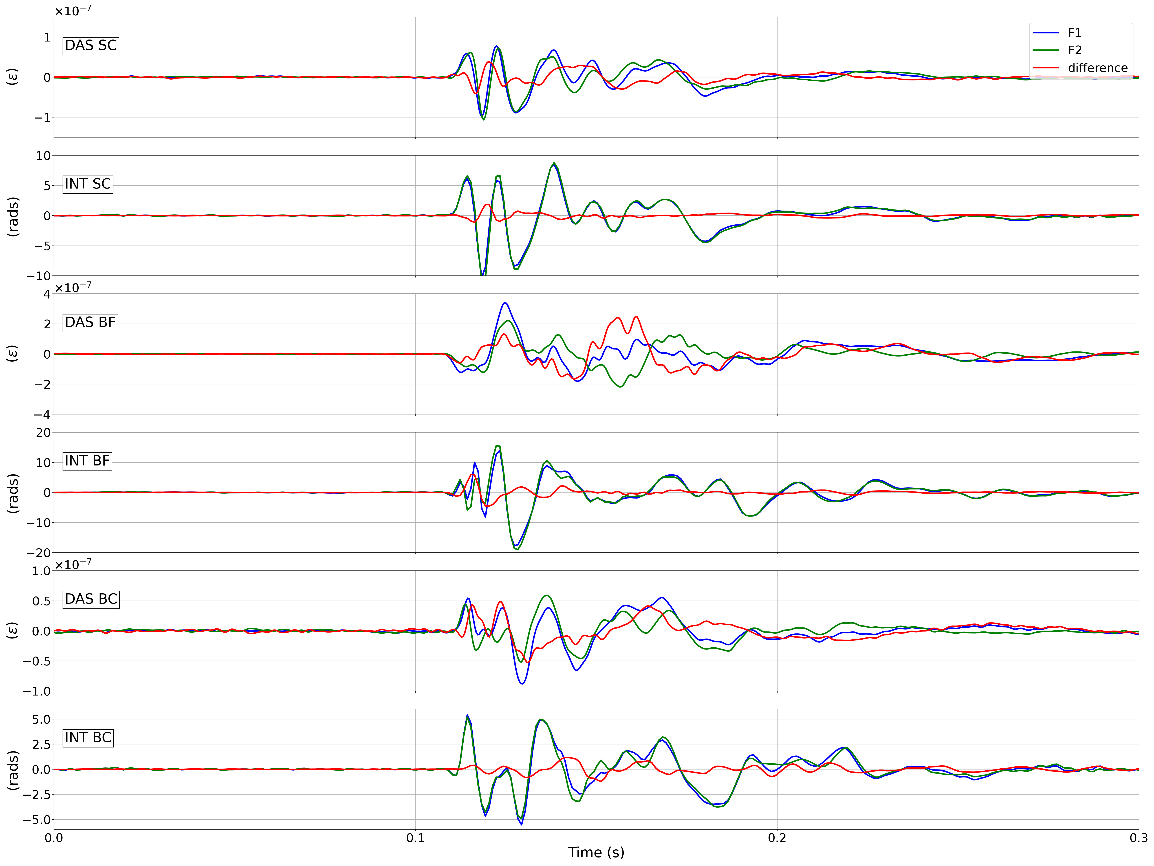


Strike 2


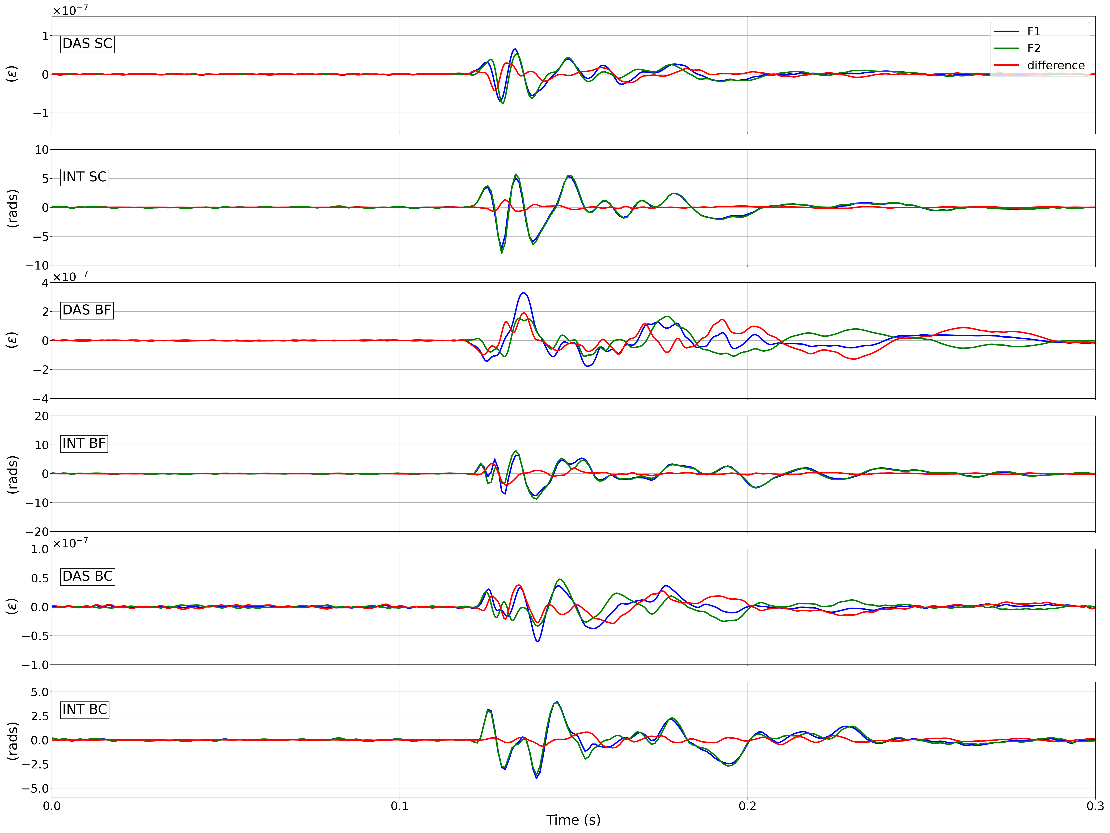


Strike 3


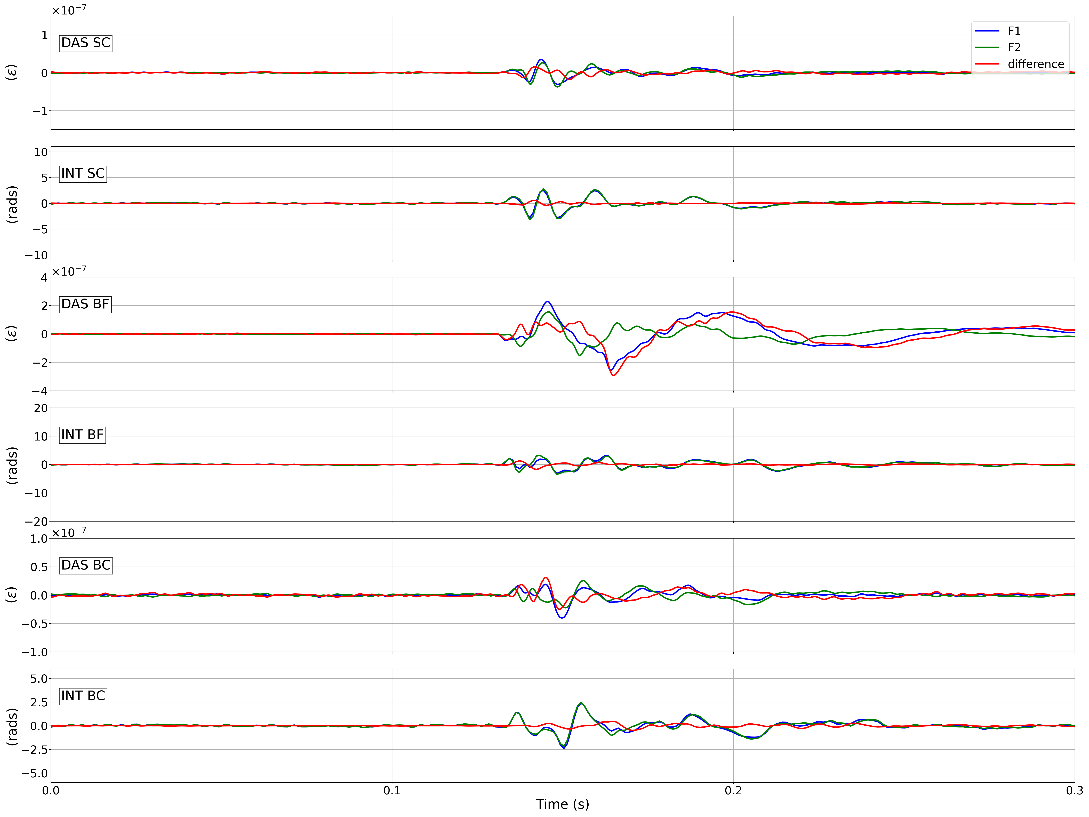


**6. JF on and off sand**


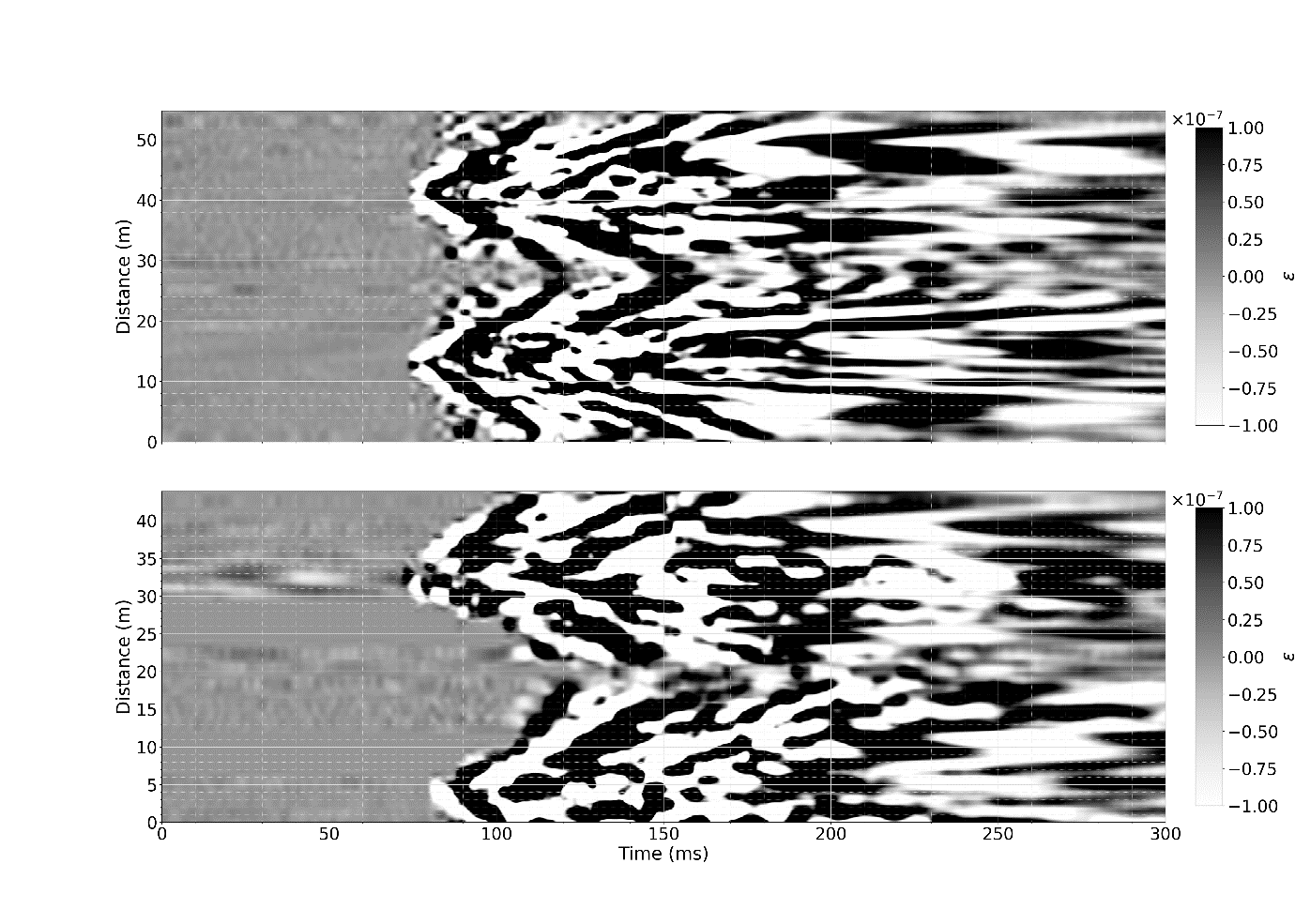


**Figure SM6**. Greyscale plots showing the DAS measurements on the JF (top panel) and the EF (bottom panel). Note the lack of a fast wave on the EF as opposed to the JF.

**7. Effect of helical fibre geometry within cables**

The helical geometry of the fibres within the small cable presents an additional complexity as it creates simultaneous extensional and compressional action upon the fibre within one gauge length. Part of the energy from any source direction may then cancel and the polarities of the amplitudes reverse depending on the wrapping angle of the fibre around the central scaffold as (see figure 11 from [22]). Polarity-flipping was also observed from a single source in a laboratory setting by Masoudi et al. [33], and indeed we see similar checkerboard-style behaviour in the DAS data for the SD cable in Fig. 3. Fibre wound in a helical arrangement can also reduce sensitivity, depending on the wrapping angle [34,35]. Hasani & Drijkoningen [22] observe a power decrease of 8-12dB from observations and models of HWF when compared against straight fibres.

**Apparent velocities of offset sources**

The velocities quoted in the main text are calculated from the moveout of the geophone arrays (Fig. 4). The inline shots can take the gradient of the time-distance moveout lines as they are but the arrivals on the geophones closest to an offset source will be hyperbolic until the azimuth between the geophone line and the source location is small. We can pick first arrivals with an accuracy of 1 ms, from those geophones where the initial curvature is negligible as our offset sources are sufficiently close to the array that we can quickly discern linear moveout.

Methods comparable to those from reflection seismology can be used for further sources but we see comparable velocities between inline and small-offset sources within an acceptable range. Uncertainties from assuming a true source time (that we unfortunately could not record) and taking the travel time picks from data sampled at 1kHz lead to a maximum velocity less than 100 ms^-1^ greater than our calculated velocity – still far lower than the fast wave velocities observed.

We consider this to be an acceptable range for the velocity calculations as geological interpretation of the subsurface relies on ranges of possible velocities depending on porosity, fluid saturation and anisotropy of the underlying geology.
